# Microbes Attaching to Endoparasitic Phytonematodes in Soil Trigger Plant Defense Upon Root Penetration by the Nematode

**DOI:** 10.3389/fpls.2020.00138

**Published:** 2020-02-25

**Authors:** Olivera Topalović, Sandra Bredenbruch, A. Sylvia S. Schleker, Holger Heuer

**Affiliations:** ^1^ Department of Epidemiology and Pathogen Diagnostics, Julius Kühn-Institut—Federal Research Centre for Cultivated Plants, Braunschweig, Germany; ^2^ Rheinische Friedrich-Wilhelms-University of Bonn, INRES—Molecular Phytomedicine, Bonn, Germany

**Keywords:** root-knot nematode, MAMP, pathogen-triggered immunity, induced systemic resistance, suppressive soil, antagonistic bacteria

## Abstract

Root-knot nematodes (*Meloidogyne* spp.) are among the most aggressive phytonematodes. While moving through soil to reach the roots of their host, specific microbes attach to the cuticle of the infective second-stage juveniles (J2). Reportedly, the attached microorganisms affect nematodes and reduce their performance on the host plants. We have previously shown that some non-parasitic bacterial strains isolated from the cuticle of *Meloidogyne hapla* in different soils affected J2 mortality, motility, hatching, and root invasion. Here we tested whether cuticle-attached microbes trigger plant defenses upon penetration of J2. In *in vitro* assays, *M. hapla* J2-attached microbes from a suppressive soil induced pathogen-associated molecular pattern-triggered immunity (PTI) in tomato roots. All tested PTI-responsive defense genes were upregulated after root invasion of J2 with attached microbes, compared to surface-sterilized J2, particularly the jasmonic acid-mediated PTI marker genes *TFT1* and *GRAS4.1*. The strain *Microbacterium* sp. K6, that was isolated from the cuticle, significantly reduced root invasion when attached to the J2. Attached K6 cells supported plant defense and counteracted suppression of plant basal defense in roots by invaded J2. The plant response to the J2-attached K6 cells was stronger in leaves than in roots, and it increased from 1 to 3 days post inoculation (dpi). At 1 dpi, the plant responded to J2-attached K6 cells by ameliorating the J2-triggered down-regulation of defense genes mostly in roots, while at 3 dpi this response was systemic and more pronounced in leaves. In a reactive oxygen species (ROS) assay, the compounds released from J2 with attached K6 cells triggered a stronger ROS burst in tomato roots than the compounds from nematodes without K6, or the metabolites released from strain K6 alone. Leaves showed a 100 times more sensitive response than roots, and the metabolites of K6 with or without J2 induced strong ROS bursts. In conclusion, our results suggest the importance of microorganisms that attach to *M. hapla* in suppressive soil, inducing early basal defenses in plants and suppressing nematode performance in roots.

## Introduction

Plant-parasitic nematodes feed on many crops worldwide and, if not maintained below the damage threshold in soil, they can cause enormous yield losses with an estimation of 100 billion dollars a year (Coyne et al., 2018). The genus *Meloidogyne* [root-knot nematodes (RKN)] has gained substantial attention in science based on the damage it causes to plants (Jones et al., 2013). The RKN infective second-stage juveniles (J2) enter the host roots at the elongation zone and, upon moving through the apoplast, inject effector proteins through a hollow stylet into the host cells to manipulate their functions and suppress plant defense (Sijmons et al., 1991; Gheysen and Mitchum, 2011). In the vascular cylinder, the effectors induce a repeated mitosis of the surrounding cells without cytokinesis, resulting in the formation of 5 to 7 giant cells that become permanent feeding sites of the nematode (Gheysen and Mitchum, 2011; Siddique and Grundler, 2018). Up to the formation of permanent feeding sites, RKN J2 do not cause huge damage during the migration phase in roots (Williamson and Hussey, 1996). It was shown that, unlike cyst nematodes, RKN do not activate damage-associated molecular pattern-triggered immunity (Shah et al., 2017), but cause expression of certain defense genes (Williamson and Hussey, 1996; Teixeira et al., 2016). Thus, they employ insidious strategies to reduce their recognition by plant. However, the life cycle of RKN can be disrupted by beneficial soil microorganisms that induce plant defenses (Adam et al., 2014a). Root border cells and root exudates play a crucial role in shaping the microbial communities in the rhizosphere, eventually resulting in a positive plant-soil feedback (Bertin et al., 2003; Bais et al., 2006; Doornbos et al., 2012; De-la-Peña and Loyola-Vargas, 2014; Ma et al., 2017). Evidence is accumulating that certain beneficial microorganisms suppress plant-parasitic nematodes by inducing systemic resistance (ISR) in plants (Reitz et al., 2000; Munif et al., 2001; Siddiqui and Shaukat, 2004; Dababat and Sikora, 2007; Adam et al., 2014b; Selim et al., 2014; Martínez-Medina et al., 2017b; Elhady et al., 2018; Kang et al., 2018; Silva et al., 2018; Topalović and Heuer, 2019). In the initial phase of microbially induced plant defense the pattern recognition receptors localized on the plant cell membranes recognize molecular structures on the microbe/pathogen surface that are referred to as microbe/pathogen-associated molecular patterns (MAMP/PAMP) (Jones and Dangl, 2006). This leads to the activation of PAMP-triggered immunity (PTI) in plants, i.e. rapid release of reactive oxygen species (ROS) (Lamb and Dixon, 1997), callose deposition on the cell walls (Millet et al., 2010), mitogen-activated protein kinase (MAPK) signaling (Wu and Baldwin, 2010), and multiple transcriptional changes (Campos et al., 2014; Coninck et al., 2015). Phytohormones such as jasmonic acid (JA), ethylene, and salicylic acid (SA) orchestrate the plant defense responses often in an antagonistic manner (Pieterse et al., 2012), but their crosstalk seems to be essential to induce systemic resistance by beneficial microorganisms (Wees et al., 2008; Martínez-Medina et al., 2013; Martínez-Medina et al., 2017a). For instance, Martínez-Medina et al. (2017a) reported that the nematode-antagonistic fungus *Trichoderma harzianum* T-78 activates a higher expression of SA marker genes in the early stages of tomato infection by the RKN *Meloidogyne incognita*, but also inhibits the suppression of JA marker genes that are expressed in the absence of the fungus.

The idea for the current study came from the previous finding that a specific microbial portion attached to the cuticle of infective nematode stages in soils may induce a varying degree of suppressiveness against plant-parasitic nematodes (Adam et al., 2014b; Elhady et al., 2017). Recently, we have isolated bacterial strains attached to the J2 of the RKN *Meloidogyne hapla* in different soils, and showed their antagonistic effects against nematodes with respect to mortality, motility, and root invasion (Topalović et al., 2019). One of the soils that we used for the J2 incubation to isolate attached microbes was obtained from a glasshouse of Geisenheim University, in the southeastern part of Germany. Remarkably, the extracted microbiomes from this soil suppressed performance of two endoparasitic nematode species, *M. hapla* and *Pratylenchus neglectus*, on tomato plants (Topalović et al., 2020). In a split-root experiment with *M. hapla,* the microbiomes reduced nematode invasion by ISR in plants, while the progeny was reduced by ISR and a direct antagonism.

Based on the above, we aimed to test the hypothesis that nematode-attached microbes might induce defense reactions in plants when *M. hapla* penetrated the root. Either the associated microbes would release compounds from the cuticle of the nematode that would act as PAMP, or the J2-transmitted microbes are recognized by plant receptors to trigger PTI defense responses. To test these hypotheses, we compared the expression profiles of several PTI-responsive genes in tomato roots inoculated with J2 of *M. hapla* with and without attached microbes from the suppressive Geisenheim soil. In addition, we studied the ROS burst of tomato plants in response to J2 of *M. hapla* and their associated bacterial isolates. Finally, we studied the ability of J2-attached bacterial cells to induce changes in expression of PTI-like genes in tomato plants and to suppress J2 invasion into roots. Our results contribute to understanding the biological importance of the microbial attachment to the infective stages of endoparasitic nematodes and the ability of the nematode-attached microbiome to accelerate defense responses of the plant against further nematode attack.

## Materials and Methods

### Surface-Sterilization of *M. hapla* J2 and Incubation in Soil Suspension

*M. hapla* J2 were extracted and surface-sterilized as previously described (Topalović et al., 2019). Briefly, eggs were extracted from egg masses on tomato roots using 1.5% sodium-hypochlorite and collected on 20 µm sieves. The eggs were transferred to a modified Baermann tray to allow hatching of J2 over a seven-day period. Freshly hatched J2 were collected every day and stored at 4°C until surface sterilization. J2 were surface sterilized using 0.02% HgCl_2_ for 2 min, washed with sterile tap water, and incubated for 4 h in an antibiotic solution of 200 mg l^−1^ streptomycin sulfate, 25 mg l^−1^ rifampicin, and 10x Cell Culture Guard (PanReac AppliChem). After incubation, the J2 were extensively washed on a 5-µm sieve with sterile tap water. They were incubated for 2 days at room temperature to recover after surface sterilization.

To allow microbial attachment to nematodes, the *M. hapla* J2 were incubated in soil suspensions of a *M. hapla*-suppressive soil from Geisenheim (sandy clay soil with 2.7% humus, pH 7.4, 49°59'01” N, 7°57'25.5” E; Topalović et al., 2020). The soil was sieved through a 1 mm sieve and 30 g of sieved soil was blended with 120 ml of sterile tap water (10 g of soil with 2 × 20 ml) for 1 min at a high speed (Stomacher^®^80, LAB SYSTEM). The suspension decanted from Stomacher bags, was then centrifuged for 5 min at 500 *g* to remove soil particles. The supernatant containing released soil microbes was sieved through a sterile 5-µm sieve to remove native nematodes and retain larger particles. The flow through was centrifuged for 10 min at 5,000 *g* and the pellet was resuspended in 30 ml sterile tap water. In 3 ml of this suspension containing 10^7^ CFU ml^−1^ bacteria and 10^4^ CFU ml^−1^ fungi, 2,000 surface-sterilized J2 were incubated in 6-well plates overnight at 20°C on a shaker at 30 rpm. As a control, J2 were incubated in 3 ml of sterile tap water. Both treatments were replicated 10 times. After incubation, loosely attached microbes were removed by washing the J2 on sterile 5-µm sieves with 20 ml of sterile tap water. The soil suspension was plated on R2A media (Merck, Germany) supplemented with 100 mg l^−1^ cycloheximide and potato dextrose agar (Merck, Germany) supplemented with 50 mg l^−1^ streptomycin-sulfate, to determine the number of cultivable bacteria and fungi, respectively. The R2A plates were kept at 28°C for 2 days and the potato dextrose agar plates at room temperature for seven days before counting the colony forming units.

### Incubation of *M. hapla* J2 With *Microbacterium* sp. K6

The J2 were surface sterilized as described above. The isolation and identification of *Microbacterium* sp. isolate K6 were done previously (Topalović et al., 2019). Bacteria were grown in liquid LB media at 28°C overnight, short-spinned for 30 s and resuspended in sterile tap water. Around 2,000 J2 were incubated in 1 ml of the bacterial suspension (1.9 · 10^7^ CFU) in 1.5 ml microtubes overnight. Loosely attached bacterial cells were removed by washing J2 on 5-µm sieves with 20 ml of sterile tap water.

### Inoculation of Nematodes to Tomato Plants Growing in Sterile Media

Tomato seeds (*Solanum lycopersicum* cv. ‘Moneymaker') were surface sterilized using 70% ethanol for 1 min and 2.5% NaOCl for 3 min. They were washed with sterile water and air-dried. The seeds were planted in MS media (Murashige Skoog, Duchefa Biochemie) supplemented with 2% Gelrite (Duchefa Biochemie) at pH 5.7. The plates with tomato seeds were kept in a climate chamber at 20°C with a 16 h photoperiod (Panasonic Versatile Environmental Test Chamber MLR-352H). To study if J2-attached microbes from soil suspension trigger expression of PTI-responsive genes in tomato roots, 350 J2 either with or without attached microbes were inoculated in a 10-µl drop at the root base and tip. Control plants were inoculated with 10 µl of sterile tap water. The plates (10 biological replicates) from each treatment were kept in a climate chamber for 3 days to let the J2 penetrate the roots. Similarly, to study whether attached bacterial cells of the strain K6 trigger expression of PTI-responsive genes in tomato leaves and roots, plants were inoculated with 350 J2 with or without attached bacterial cells as described above. In this experiment, each treatment was replicated twenty times and kept in a climate chamber for 1 or 3 days, respectively. Additional four replicates in each treatment were used to microscopically determine the number of invaded J2 in roots at 7 days post inoculation (dpi) after staining with acid fuchsin (Sigma Aldrich). For both experiments, small labeled bags were prepared from aluminium foil to quickly sample and freeze plants for RNA extraction. Using forceps, whole plants were carefully removed from the plates and adhering Gelrite was swiftly removed with a paper tissue. The plants were transferred to the bags, which were closed, immersed in liquid N_2_, and then stored at −80°C until RNA extraction.

### RNA Extraction and cDNA Synthesis

Frozen roots and leaves were transferred to separate 2 ml microtubes and pulverized with a 5 mm metal bead in a TissueLyser II (Qiagen) twice for 20 s at the highest speed (30 Hz), keeping samples frozen in a pre-cooled block. Total RNA from plants was extracted using the FastRNA Pro Green Kit (MP Biomedicals). Briefly, 1 ml of RNApro solution was added to each tube and mixed well by inverting the tubes and pipetting to inactivate RNases and start chemical cell lysis. The suspension was transferred to a bead-beating tube and cells were mechanically lysed in a FastPrep-24 instrument (MP Biomedicals) for 35 s at speed level 6.0 m s^−1^. After centrifugation at 4°C for 5 min at 14,000 *g*, 800 µl of the supernatant was extracted with 300 µl chloroform by vortexing for 10 s and incubation at room temperature for 5 min. After phase separation at 14,000 *g* for 5 min at 4°C, the aqueous phase was transferred to a new tube. RNA was precipitated by adding 500 µl cold absolute ethanol, inverting the tubes five times and incubation at −20°C for 30 min. The RNA was pelleted by centrifugation at 4°C for 15 min at 14,000 *g* and washed with 500 µl cold RNase-free 70% ethanol. The RNA was resuspended in 30 µl of RNase-free water (Invitrogen) and kept at −80°C until use. Remaining traces of DNA were removed from aliquots of 1 µg RNA by DNase I digestion followed by DNase inactivation and removal, using the DNA-*free* DNA Removal Kit (ThermoFisher Scientific). Purified RNA was reverse transcribed using Superscript IV according to the manufacturer's instructions (ThermoFisher Scientific). Briefly, 8 µl RNA (ca. 1 µg) were incubated with 1 µl 50 µM oligo dT(23), 1 µl dNTP (10 mM each) and 3 µl nuclease-free water at 65°C for 5 min and on ice for at least 1 min. Superscript IV (200 U in 1 µl), 4 µl 5× SSIV Buffer, 1 µl 100 mM DTT, and 1 µl RNase OUT were added and cDNA synthesized at 55°C for 10 min. Samples were heated to 80°C for 10 min and stored at −20°C.

### Real-Time qPCR Analysis of Defense Gene Expression

Real-time amplifications were performed using a CFX Connect Real Time Detection System (Bio-Rad) in 20 µl reactions containing Standard *Taq* Reaction Buffer (New England BioLabs), 0.25 mM MgCl_2_, 0.2 mM of each dNTP, 2× EvaGreen (Biotium), 1 µM of gene specific primers (**Table 1**), and 0.5 U Hot Start *Taq* DNA Polymerase (New England BioLabs). Thermocycles were as follows: initial denaturation at 94°C for 5 min, 40 cycles of a denaturation step at 94°C for 20 s, annealing step at 60°C for 30 s, an extension step at 68°C for 30 s, and 80°C for 30 s. The fluorescence was read at the 80°C step of each cycle. After each run, a melting curve was generated between 65° and 95°C. Ct values of defense genes were normalized to the ubiquitin gene expression, and their relative expressions in each sample were determined against gene expressions in control plants without nematodes, using the 2^–ΔΔCt^ method (Pfaffl, 2001).

**Table 1:** Primer pairs used in this study.

Gene	Forward primer (5′-3′)	Reverse primer (5′-3′)	Reference
*PR1a1*	CTGGTGCTGTGAAGATGTGG	TGACCCTAGCACAACCAAGA	Harel et al., 2014
*PDF1.2*	GGCTAGCAAAATCACTTTCTGTG	CATGATCCTTATTTTTGCACCA	Tran et al., 2017
*GRAS4.1*	TTCGAATCCCCTGCTTCCAT	CCAGTTGGTGAATTGCTGCT	This study
*MPK1*	GCTGACAGATTGTTGCAGGT	TCCACCCCATAAAGATACATCA	Kandoth et al., 2007
*WRKY28*	ACAGATGCAGCTACCTCATCCTCA	GTGCTCAAAGCCTCATGGTTCTTG	Taylor et al., 2012
*TFT1*	GCCTCGTCCATCTGCTCCTG	GAATGCATCAGAAAAAGCATGCAG	Taylor et al., 2012
*PTI5*	ATTCGCGATTCGGCTAGACATGGT	AGTAGTGCCTTAGCACCTCGCATT	Taylor et al., 2012
*Ubiquitin*	GTGTGGGCTCACCTACGTTT	ACAATCCCAAGGGTTGTCAC	Bhattarai et al., 2008
*Meloidogyne* 18S rRNA	AAGATATCTGGTTGATCCTGCCTGA	GTTCAAAGTAAACTTGCAAYGMCTG	This study

### Real-Time qPCR Analysis of *Meloidogyne* 18S rRNA in cDNA of Roots

Relative quantification of the 18S rRNA gene of *M. hapla* inside tomato roots infested with J2 with and without attached K6 strain were determined using 5 µl of cDNA samples. A 750 bp fragment of the *M. hapla* 18S rRNA of invaded J2 in roots was amplified in real-time qPCR using a CFX Connect Real Time Detection System (Bio-Rad) in 25 µl reactions containing Standard *Taq* Reaction Buffer (New England BioLabs), 0.25 mM MgCl_2_, 0.2 mM of each dNTP, EvaGreen (Biotium), 1 µM of *Meloidogyne* spp. specific primers Melo1f (5′-AAGATATCTGGTTGATCCTGCCTGA-3′) and Melo723r (5′-GTTCAAAGTAAACTTGCAAYGMCTG-3′), 0.625 U Hot Start *Taq* DNA Polymerase (New England BioLabs). After initial denaturation at 94°C for 2 min, fragments were amplified in 40 cycles with a denaturation at 94°C for 20 s, annealing at 57°C for 20 s, extension at 72°C for 45 s, and fluorescence detection at 80°C for 30 s. Melting curves were generated between 65° and 95°C. Ct values were normalized to the ubiquitin gene expression, and 18S rRNA of J2 with attached bacterial strain K6 were determined relative to 18S rRNA of J2 without K6 using the 2^–ΔΔCt^ method (Pfaffl, 2001).

### ROS Burst Assay

The *Microbacterium* sp. isolate K6 and *Acinetobacter* sp. isolate E1 were obtained in a previous study (Topalović et al., 2019). Around 10,000 surface sterilized J2 of *M. hapla* were incubated in 200 µl suspensions of the strains K6 or E1 (10^7^ CFU ml^−1^), or 200 µl of sterile tap water in 1.5 ml microtubes, at room temperature overnight without shaking. The bacterial suspensions were prepared as described previously (Topalović et al., 2019). The supernatants were sterile-filtered the following day and served as the surface-released compounds from the J2 (SC) with and without bacterial cell-free supernatants. The J2-bacteria pellets were suspended in 200 µl of sterile tap water to get suspensions of live J2 with and without the living bacteria.

To measure ROS burst we adapted previously published methods (Prince et al., 2014; Mendy et al., 2017). Briefly, the cotyledon leaves were excised from 12-days-old tomato plants and incubated in ddH_2_O in the dark for 12 h. The leaf samples were then transferred to a 96-well plate that contained 80 µl of 0.1 µM luminol derivate 8-amino-5-chloro-2,3-dihydro-7-phenyl-pyrido[3,4-d] pyridazine sodium salt (L-012, Wako Chemicals). After another hour of incubation in the dark, 20 µl of 20 µg ml^−1^ horseradish peroxidase was added to each well followed by 50 µl elicitor, with 1 µM flagellin peptide (Flg22) as positive and sterile tap water as negative controls. The elicitor treatments were as follows: (a) Live J2, (b) SC, (c) Live J2 + K6 cells, (d)SC + K6 cell-free supernatant, (e) Live J2 + E1 cells, (f) SC + E1 cell-free supernatant. The assay was repeated three times with three technical replicates each. Relative light units of luminescence were measured in a 96-well microplate reader (Infinite^®^ 200 PRO; TECAN) over 120 min. The data were analyzed using instrument software and Microsoft Office Excel.

### Statistical Analysis

Fold changes in expression of each defense gene after root penetration of J2 with or without attached microbes, as related to expression without nematodes, were log-transformed to better fit linear models (smaller AIC). Generalized linear models were applied using the statistical software SAS 9.4. Class variables were MICROBES (1, 0) and GENE (*GRAS4.1*, *MPK1*, *PDF1.2*, *PR1a1*, *TFT1*, *WRKY28*) for the experiment with attached microbiome from Geisenheim soil, and additionally DPI (1, 3) and TISSUE (leaf, root) for the experiment with the attached bacterial strain K6. A *post hoc* Tukey-test was done to compare fold changes in expression of defense genes in plants invaded by J2, with or without attached microbes.

## Results

### J2-Attached Microbes From Geisenheim Soil Trigger Expression of PTI-Responsive Genes in Tomato Roots

**Figure 1:**
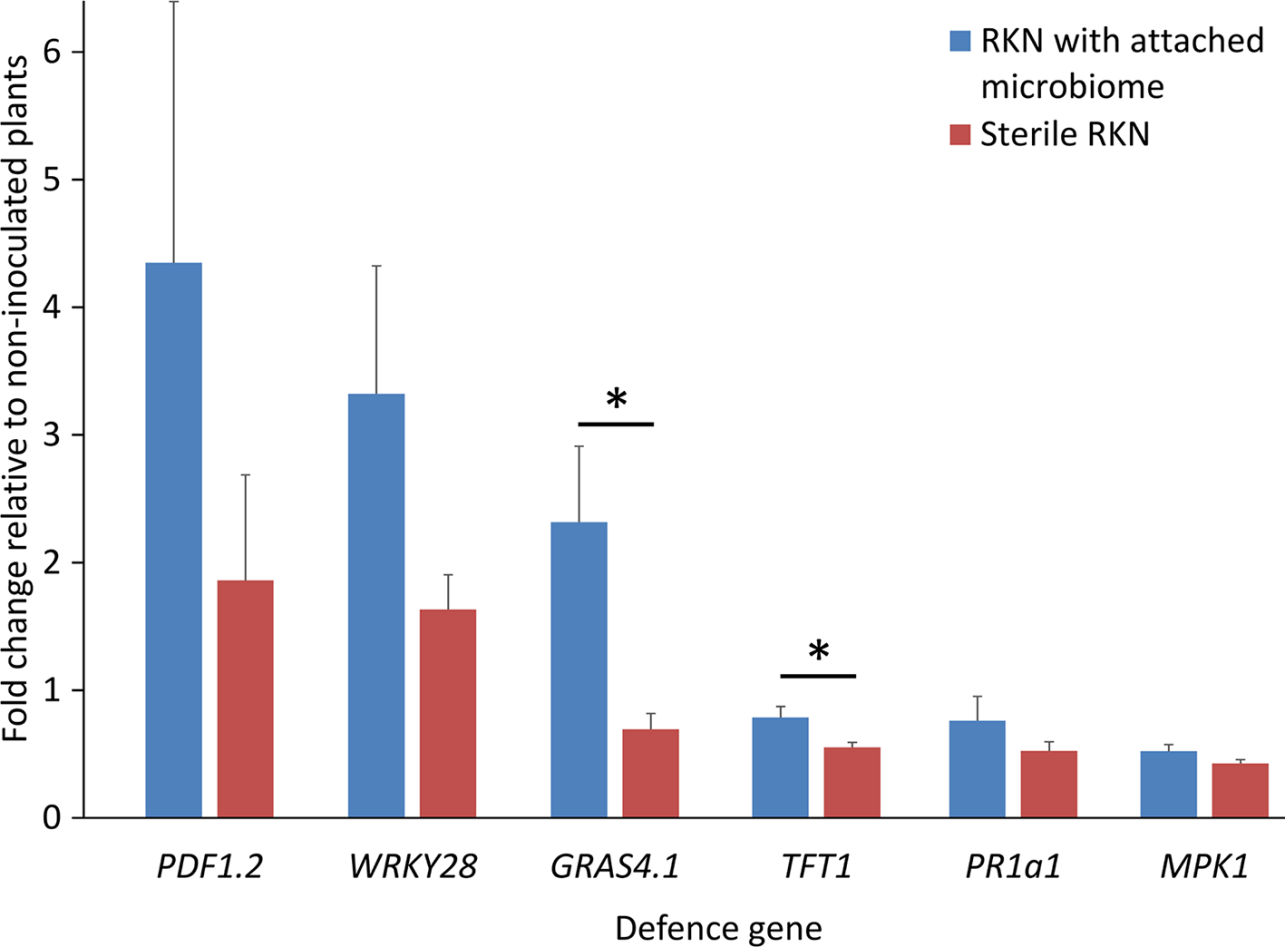
Expression profiles of PTI-like genes in tomato roots in response to root penetrating juveniles of the root-knot nematode (RKN) *Meloidogyne hapla* with and without attached microbes from suppressive Geisenheim soil, 3 dpi. ANOVA by generalized linear models (see **Supplement Statistics 1**) revealed that the attached microbes significantly enhanced defense gene expression overall (*P* = 0.001), while the response of the various genes differed (*P* = 0.0001). Lines under an asterisk indicate significant differences between plants after invasion of nematodes with or without microbes attached to the cuticle (*P* < 0.05, Tukey test on log-transformed data). Error bars represent SE (n = 10 plants).

To study if the microbiome attaching to the pre-infective J2 of *M. hapla* in Geisenheim soil induce PTI responses in tomato plants, the J2 were incubated in microbial suspensions overnight, washed, and nematodes with or without attached microbial cells were inoculated to 2-week old tomato plants growing in MS media. The differential expression of six PTI marker genes 3 dpi was determined compared to their expression in roots without invaded nematodes. All tested PTI marker genes were expressed in the roots infested with both, clean juveniles and juveniles carrying the attached soil microbes. However, the J2 with attached microbes triggered PTI responses higher than those of the surface-sterilized J2 alone (**Figure 1**). Overall, ANOVA revealed a significant effect of attached microbes on defense gene expression (*P* = 0.001). The response of the genes to J2 differed (*P* = 0.0001), while the interaction of GENE * MICROBES was not significant (*P* = 0.55). The average up-regulation of the plant defense genes by J2 with attached microbes was two-fold. However, data showed that J2 without attached microbes managed to down-regulate most of the defense genes (*GRAS4.1*, *MPK1*, *PR1a1*, *TFT1*), and that attached microbes counteracted this. The transcription factor *GRAS4.1* was significantly up-regulated 3 dpi when the roots were invaded by J2 with microbes attached to the cuticle, while in the absence of attached microbes this gene was repressed by the J2 (*P* = 0.002, Tukey test). Conversely, the transcription factor *TFT1* was down-regulated in both treatments, but the down-regulation was ameliorated by microbes attached to J2 (*P* = 0.035, Tukey test). Although microbial attachment to the J2 of *M. hapla* in Geisenheim soil led to the up-regulation of both the transcription factor *WRKY28* and the JA-regulated *PDF1.2* defensin gene, this was not statistically supported. Mitogen-activated protein kinase 1 gene (*MPK1*) and the marker gene of the SA pathway (*PR1a1*) were both down-regulated with minor differences between the two treatments. Independent soil sampling and incubation of J2 confirmed the effect of J2-attached microbes on defense gene expression. However, *TFT1* was significantly up-regulated by the attached microbes while down-regulated without microbes (**Supplementary Figure S1**).

### Attachment of Bacterial Isolate K6 to *M. hapla* J2 Affected Root Invasion and Expression of PTI Marker Genes

**Figure 2:**
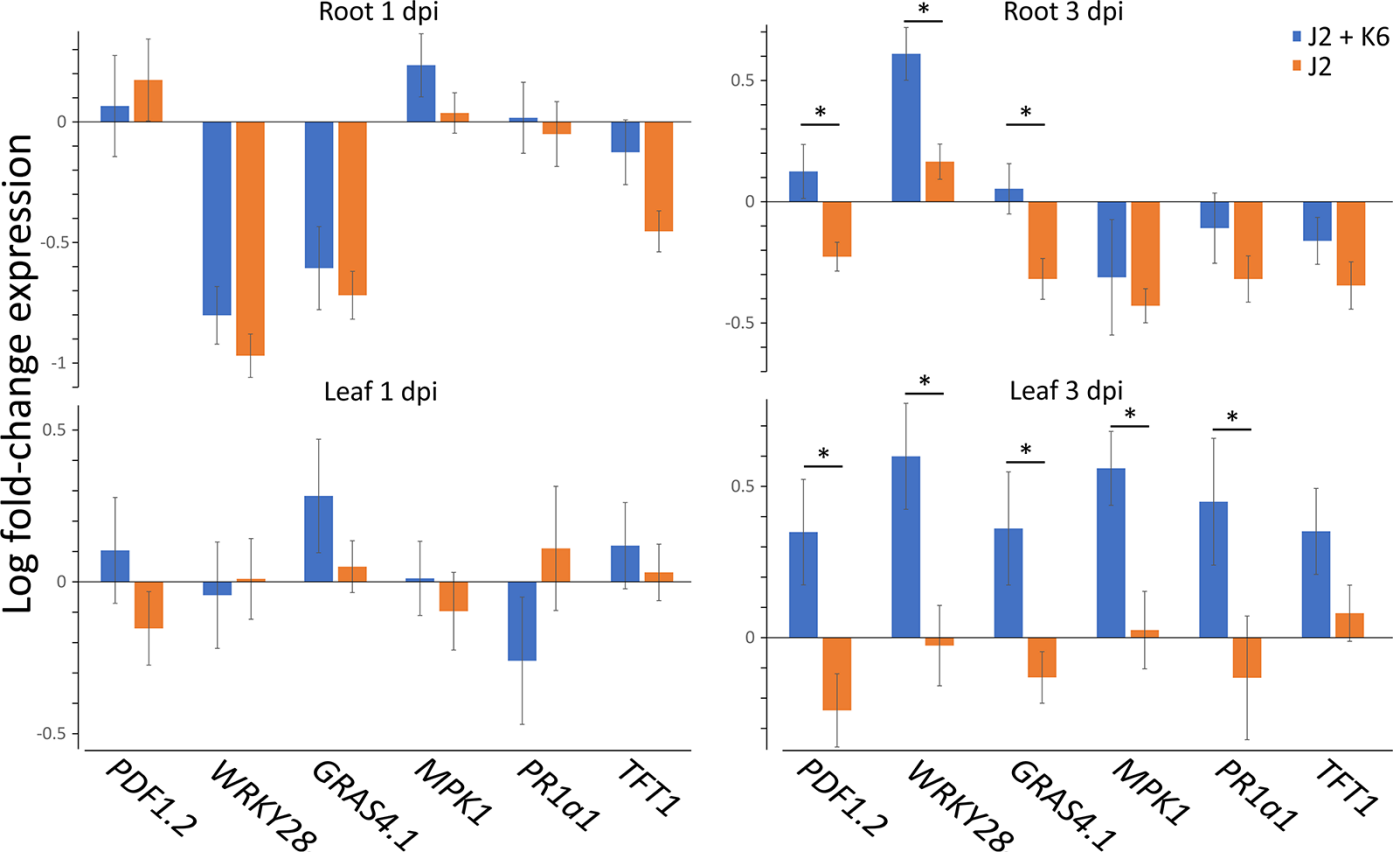
Expression profiles of PTI genes in leaves and roots of tomato plants in response to *M. hapla* J2 with and without attached *Microbacterium* sp. K6. Defense gene expression of invaded plants relative to not inoculated plants, as determined in cDNA by Real-Time PCR, was normalized for ubiquitin transcripts and *M. hapla* 18S rRNA in roots, using the 2^–ΔΔCt^ method. Bars show means of log-transformed fold-changes in gene expression, one and three days after J2 inoculation to the roots (dpi). ANOVA by generalized linear models (see **Supplement Statistics 2**) revealed that the attached bacteria K6 (stages: with, without) significantly enhanced defense gene expression overall (*P* < 0.0001). The factors DPI (stages: 1 dpi, 3 dpi) and TISSUE (stages: root, leaf) had significant effects on gene expression. The effect of attached K6 was more pronounced 3 dpi than 1 dpi (*P* = 0.002 for the interaction effect K6 * DPI). Error bars represent SE (n = 10 plants).

The bacterial strain *Microbacterium* sp. K6 was isolated from the nematode cuticle after baiting of J2 in Geisenheim soil suspension. The effect of this strongly attaching strain on plant defense upon root penetration of *M. hapla* J2 was investigated. The expression profiles of six PTI-responsive genes were determined in roots and leaves of 2-week old tomato plants, 1 and 3 days after root inoculation of J2 with or without attached cells of strain K6 (**Figure 2**). Defense gene expression of invaded plants relative to uninoculated plants was normalized for ubiquitin transcripts. As the number of J2 invading the root and triggering plant defenses is reduced by strain K6, the defense gene expression was also adjusted to equal *M. hapla* 18S rRNA in roots. ANOVA showed that the attached bacteria significantly enhanced defense gene expression (*P* < 0.0001). At 1 dpi, four of the defense genes were down-regulated in roots by invading J2 (**Figure 2**). Attached K6 resulted in less down-regulation and in significant up-regulation of the mitogen-activated protein kinase 1 gene (*MPK1*) in roots. In leaves at 1 dpi, only a weak response of defense genes to J2 invasion in roots was detectable. J2-attached K6 resulted in a slightly higher expression of four of the defense genes, but a down-regulation of *PR1a1*. At 3 dpi, all six defense genes showed higher expression in roots and leaves in response to J2 with attached K6 compared to surface-sterilized J2. The attached bacteria caused a significant up-regulation of the PTI-genes in leaves and of the transcription factor *WRKY28* in roots, while surface-sterilized J2 kept PTI defense at a low level (**Figure 2**). *WRKY28* was down-regulated 1 dpi and up-regulated 3 dpi in both treatments, while the K6 cells significantly increased the up-regulation of this gene (*P* < 0.0001; Tukey test). In the ANOVA, the factors DPI and TISSUE had significant effects, and the effect of attached K6 was more pronounced 3 dpi than 1 dpi (*P* = 0.002 for the interaction effect K6 * DPI). Overall, the genes *WRKY28*, *GRAS4.1*, and *MPK1* in roots, and *MPK1* in leaves were significantly triggered by J2-attached bacteria (*P* < 0.05, Tukey correction).

**Figure 3:**
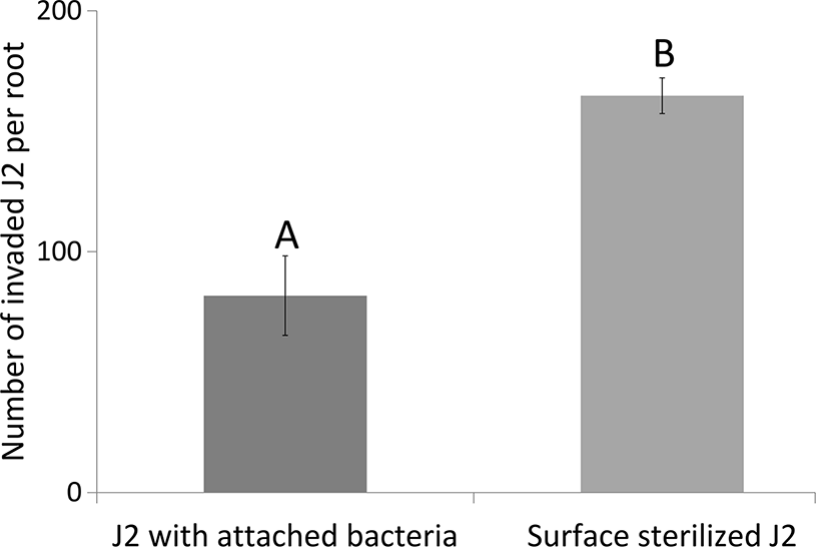
Number of invaded J2 of *M. hapla* J2 in tomato roots 7 dpi, with or without attached *Microbacterium* sp. K6. Error bars represent SD. The letters above error bars indicate a significant difference between treatments (*P* < 0.05, Tukey test; n = 4 plants).

To follow the effects of J2-attached K6 cells on nematode penetration, we determined the 18S rRNA of *M. hapla* in the same root samples that were used to study expressions of defense genes. The number of 18S rRNA increased from 1 to 3 dpi. The relative abundance of ribosomal RNA of J2 with attached K6 cells was 82% ( ± 22% SE) and 55% ( ± 15% SE), of the relative abundance of the J2 without attached bacterial cells at 1 and 3 dpi, respectively. The reduction became statistically significant at 3 dpi (*P* = 0.018, one sample t-test). With plants grown in parallel that were not sampled for RNA extraction, the number of J2 in roots was determined microscopically 7 dpi (**Figure 3**). The nematode penetration was significantly suppressed by the attached K6 cells. The number of invaded J2 in control roots was the double of those with attached K6 cells. Thus, constantly less J2 invaded the roots within the first seven days after inoculation, when bacterial cells were attached to the cuticle.

### ROS Response of Tomato Tissue to *M. hapla* J2 With and Without Attached Bacteria

**Figure 4:**
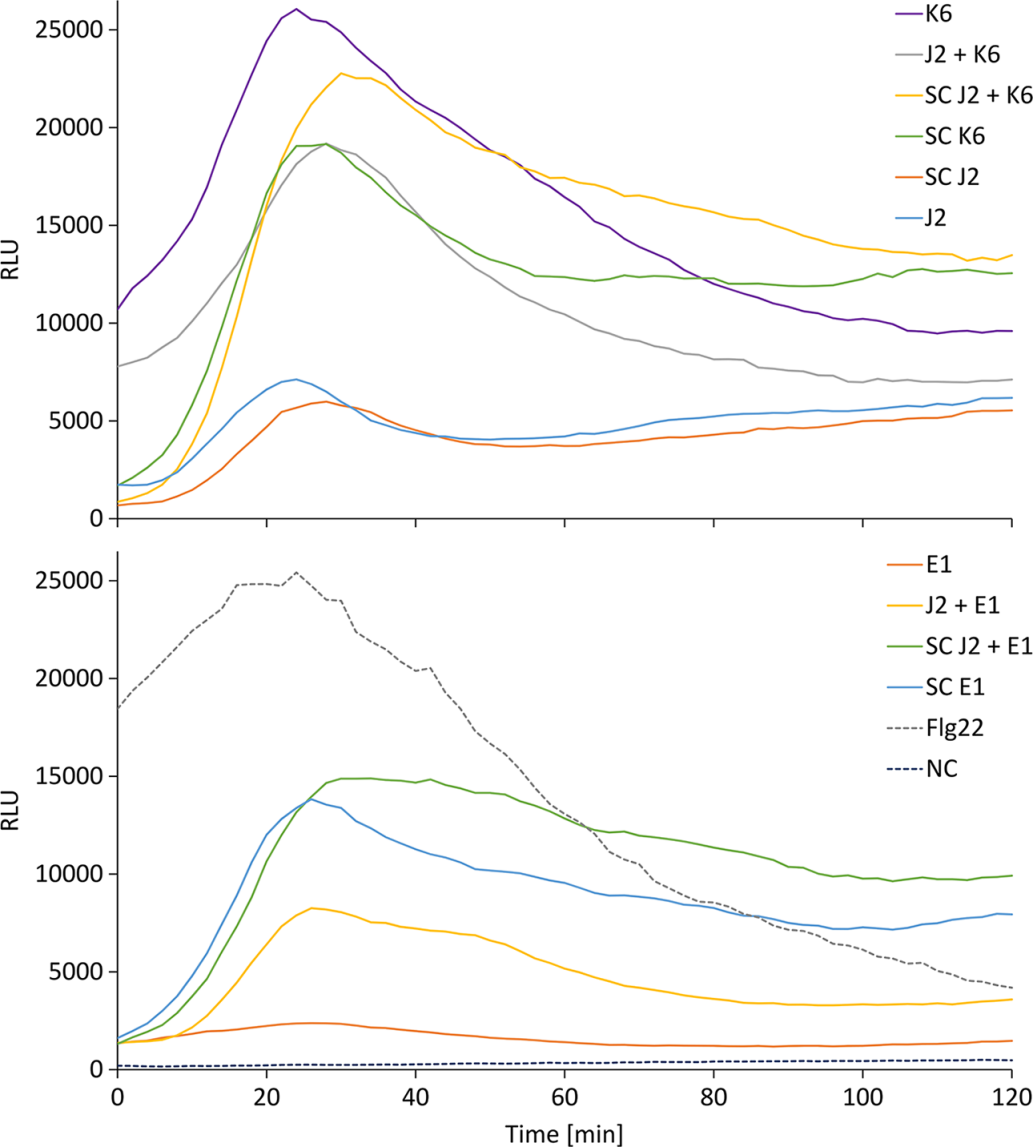
Relative luminescence units (RLU) representing the ROS burst in tomato leaves over time for the different treatments. Treatments were K6: cells of *Microbacterium* sp. K6; J2 + K6: live juveniles of *M. hapla* (J2) + cells of K6; SC J2 + K6: released compounds from J2 incubated with K6; SC K6: released compounds from K6; SC J2: released surface compounds from J2; J2: live juveniles of *M. hapla* E1: cells of *Acinetobacter* sp. E1; J2 + E1: live juveniles + cells of *Acinetobacter* sp. E1; SC J2 + E1: released compounds from J2 incubated with E1; SC E1: released compounds from E1; Flg22: positive control; NC: water as negative control. Curves show means of 6 to 12 plants.

**Figure 5:**
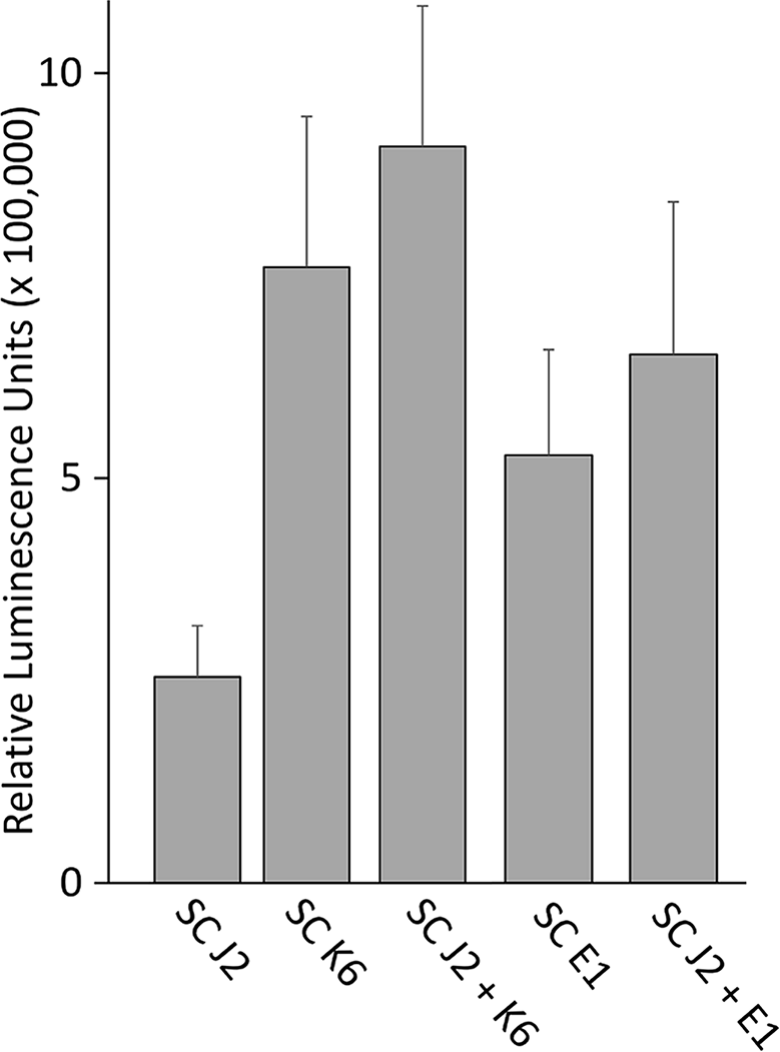
ROS burst in tomato leaves triggered by surface-released compounds (SC) from juveniles of *M. hapla* (J2), bacteria isolated from the cuticle of J2 (*Microbacterium* sp. K6, *Acinetobacter* sp. E1), or J2 incubated with these bacteria. The sum of relative luminescence units over 2 h in a ROS assay with tomato leaves is shown. Differences among treatments were not significant (*P* < 0.05, Tukey test on log-transformed data). Error bars represent SE (n = 6 to 12 plants).

**Figure 6:**
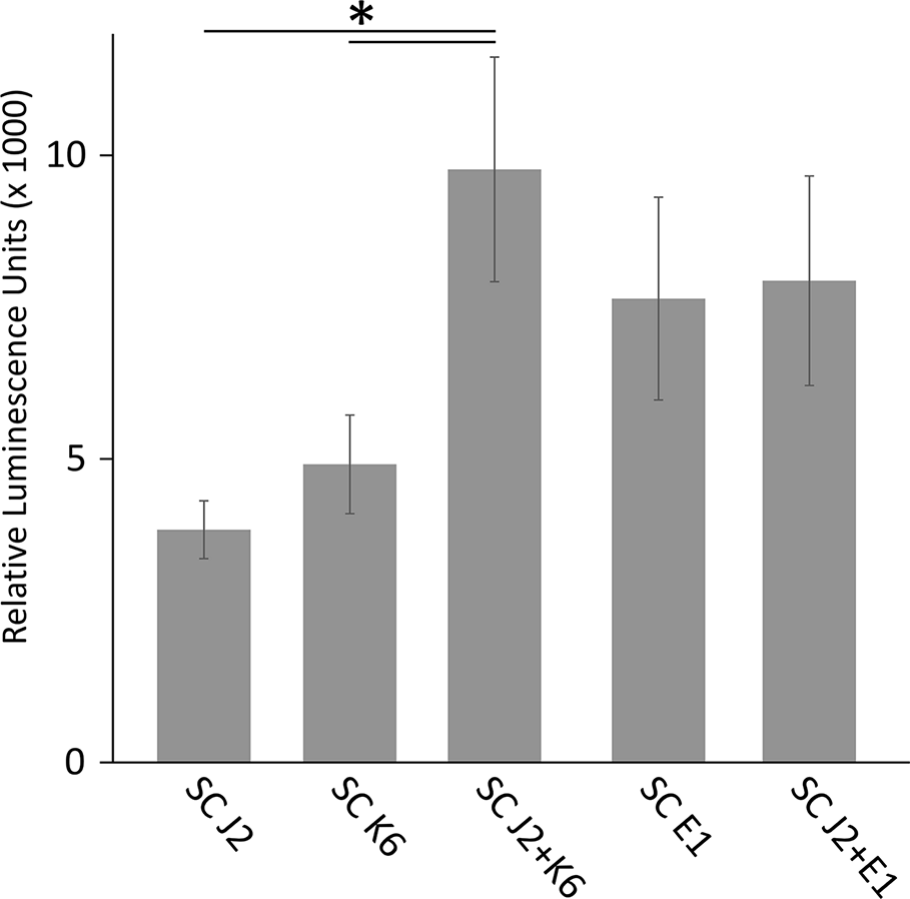
ROS burst in tomato roots triggered by surface-released compounds (SC) from juveniles of *M. hapla* (J2), bacteria isolated from the cuticle of J2 (*Microbacterium* sp. K6, *Acinetobacter* sp. E1), or J2 incubated with these bacteria. The sum of relative luminescence units over 2 h in an ROS assay with tomato roots is shown. Lines under an asterisk indicate significant differences between treatments (*P* < 0.05, Tukey test on log-transformed data). Error bars represent SE (n = 6 to 12 plants).

We tested whether PTI is triggered in tomato plants by living bacterial cells of *Microbacterium* sp. K6 and *Acinetobacter* sp. E1, or by the compounds they release, or by the compounds that are released when J2 and bacteria are incubated together. The ROS patterns of leaves and roots of tomato plants were analyzed in response to the organisms and their surface-released compounds (SC), using a luminescence assay. To produce SC, nematodes and bacteria were incubated in water overnight alone or in combination, and the supernatant containing SC was separated from the organisms by centrifugation. Although all variants, living bacterial cells as well as their SC, nematodes and their SC, the live nematodes together with bacteria, and the SC from nematodes incubated with bacteria triggered a ROS peak in leaves within 25 min, differences were observed in their respective intensities (**Figure 4**). Both the SC from J2 and living J2 triggered a clear ROS burst, which was further amplified for variants including K6 or E1. Also in the absence of *M. hapla*, compounds of both bacterial strains were capable to elicit a strong ROS burst in the leaf tissue, while only the living cells of K6 induced a similar ROS pattern (**Figure 4**). Consequently, incubation with K6 or E1 led to an intensified ROS response to the infective stage of the parasitic nematode and related SC (**Figure 4**). The compounds released from K6 and E1 were sufficient to induce a strong ROS burst in the leaf tissue (**Figure 5**). Also the SC from *M. hapla* J2 triggered a considerable ROS burst. The SC from J2 that were incubated with K6 or E1 showed a trend to induce a stronger ROS response than the SC from the respective bacteria alone, although not statistically significant (**Figure 5**). The sum of the signals from the SC of *M. hapla* J2 and the SC of bacteria were not more than the ROS signal from the combined treatments obtained in leaves (**Figure 5**). In roots, similar reactions were observed but at 100 times lower intensity (**Supplementary Figures S2**, **S3**). The SC of strain K6 triggered not significantly more ROS than SC of *M. hapla* J2, while the SC from J2 incubated with strain K6 induced a significantly stronger ROS burst in roots than SC of both organisms alone (**Figure 6**). In contrast, for strain E1 the effect of its SC did not differ from the effect of the SC from J2 + E1 that were incubated together.

## Discussion

DNA-based analysis of the microbiome associated with phytonematodes has gained attention over the recent years (Adam et al., 2014b; Cao et al., 2015; Elhady et al., 2017; Hussain et al., 2018). Characterization of microbial communities that are in a direct association with plant-parasitic nematodes in soil helps deepen the knowledge on the mechanisms that microbes deploy to antagonize these persistent plant parasites. However, previous studies mainly focused on the subtle differences in attached microbiota among different nematode stages or species and in different soils (Adam et al., 2014b; Elhady et al., 2017). Recently, we demonstrated that the soil microbiomes from a glasshouse at Geisenheim University reduced performance of *M. hapla* on tomato plants, while a split-root experiment revealed the contribution of microbially triggered ISR to this phenomenon (Topalović et al., 2020). In addition, we isolated bacterial strains from the cuticle of *M. hapla* and *Pratylenchus penetrans*, showing their antagonistic activity, but the ability of nematode-attached bacteria to induce systemic resistance in plants has not yet been investigated (Topalović et al., 2019). Our current study revealed that the early infection of tomato by *M. hapla* J2 alone resulted in the suppression of most of the analyzed PTI defense genes, but that the J2-attached microbes either ameliorated this effect, or triggered up-regulation of the genes. Plant-parasitic nematodes cause mechanical damage of the host cells, which respond by activating damage-associated molecular pattern triggered defense (Holbein et al., 2016; Shah et al., 2017). The opening wounds can also ease the entrance of pathogenic microorganisms that lead to secondary infections and disease complexes (Karimi et al., 2000; Back et al., 2002). However, the RKN are well known to hide themselves from plant recognition, since the J2 move through the apoplast until establishing permanent feeding sites (Sijmons et al., 1994). Notably, it was shown that the cell wall degradation caused by cyst nematodes, while moving through the symplast, trigger activation of polygalacturonase inhibitor proteins (PGIP) in plants, which activate damage-associated molecular pattern triggered defense by production of oligogalacturonides (Benedetti et al., 2015; Shah et al., 2017). In contrast to cyst nematodes, Shah et al. (2017) could not detect activation of PGIP in response to the RKN infection, thus pointing out the ability of RKN to suppress early recognition by the host. In addition, several studies have shown down-regulation of PTI-like genes during the early events of RKN infection (Dubreuil et al., 2007; Barcala et al., 2010; Damiani et al., 2012). In our study, J2-attached microbes from the suppressive Geisenheim soil alleviated expression of several PTI-like genes, including *GRAS4.1*, *TFT1*, *WRKY28*, and *PDF1.2*. Interestingly, the transcription factor *TFT1*, a marker of JA-mediated PTI, was down-regulated in roots infected with J2. However, the down-regulation of this gene was suppressed by the J2-attached microbes. *TFT1* belongs to the tomato 14-3-3 family of acidic, phosphopeptide-binding proteins that are involved in metabolism, signal transduction, transcription, cell cycle, development, apoptosis, and stress signaling (Bridges and Moorhead, 2004; Smith et al., 2011). Taylor et al. (2012) showed that silencing of *TFT1* resulted in increased tomato susceptibility to the pathogenic bacterium *Xanthomonas campestris* pv. *vesicatoria*, and it was also required for the expression of some other JA- and SA-mediated defense genes, including *GRAS* and *WRKY*. The inhibited repression of *TFT1* by nematode-associated microbes in our study suggests that the plant recognized the transmitted microbes upon nematode invasion. The J2-attached microbes from Geisenheim soil also significantly elevated mRNA levels of the transcription factor *GRAS4.1*. The up-regulation of the *GRAS* gene was positively correlated with the expression of *TFT1* in establishing PTI against *X. campestris* pv. *vesicatoria* (Taylor et al., 2012). *GRAS* transcription factors are involved in plant development, gibberellic acid signaling, symbiotic processes, and stress signaling (Tian et al., 2004). This gene was also up-regulated in tomato and tobacco leaves infected by non-pathogenic *Pseudomonas fluorescens* as a reporter gene for assaying PTI (Nguyen et al., 2010).

The bacterial strain K6, which belongs to the Gram-positive genus *Microbacterium*, was previously isolated from the cuticle of *M. hapla* J2. It was firmly attached to J2, with more than 4,000 CFU/J2, and showed antagonistic effects on their viability, movement, and invasion into roots (Topalović et al., 2019). However, plant-mediated effects were more important for its antagonism against *M. hapla* as shown in a split-root experiment (Topalović et al., 2019). Similar to the attachment of microbes from the microbiome of the Geisenheim suppressive soil, the J2-attached strain K6 also ameliorated the J2-induced suppression of defense genes. The expression profiles of the genes differed 1 and 3 dpi in root and leaf tissues. At 1 dpi, K6 generally reduced down-regulation of defense genes in roots, while significantly up-regulating *MPK1*. This effect was stronger 3 dpi with several up-regulated genes, including *WRKY28*, *GRAS4.1*, and *PDF1.2*. However, at 1 dpi PTI activation seemed to be limited to belowground tissues as leaves hardly responded to the J2 infection in roots. At 3 dpi, K6 had a strong systemic effect in leaves and the defense response in roots increased. A similar pattern of belowground-aboveground response was observed by Wang et al. (2019). They reported root-shoot transmission of electrical and ROS signals in response of *Arabidopsis thaliana* to *Meloidogyne incognita* 1 dpi. This led to the activation of JA synthesis in leaves. Consecutively, JA systemically triggered the defense in the roots. In our study, the overall low plant responses to J2 at 1 dpi could also be attributed to the fact that *M. hapla* J2 are slow invaders in comparison to the more aggressive RKN *M. incognita*. Martínez-Medina et al. (2017a) reported that the fungus *T. harzianum* T-78 primed local and systemic tomato root tissues during the early stages of *M. incognita* infection by accelerating activation of SA-responsive genes and inhibiting suppression of JA-responsive genes. However, studies on the expression profiles of microbially induced PTI-like genes in roots, in response to nematode infection, are still scarce. It was shown that the expression of SA marker *PR* genes differed in roots and leaves in response to the RKN, but this seemed to depend on the nematode species and timing (Hamamouch et al., 2011; Dam et al., 2018). Interestingly, the transcription factor *WRKY28* was down-regulated in our study in response to both J2 alone and the J2-attached K6 cells at 1 dpi, while it was significantly up-regulated by the K6 cells 3 dpi. Although the RKN cause less damage during the early stages of infection, up-regulation of *WRKY28* by J2 alone confirms that the RKN *M. hapla*, nevertheless, also induce expression of defense genes (Williamson and Hussey, 1996). The up-regulation of *WRKY11* and *WRKY17* genes in *A. thaliana* roots infested with *M. incognita* suggested that these transcription factors behave as positive regulators of plant defense against RKN, as both *WRKY11* and *WRKY17* mutants showed significantly higher nematode reproduction in comparison to the wild type (Teixeira et al., 2016).

We showed a strong positive correlation between the activation of PTI by J2-attached K6 cells upon nematode penetration and a reduced number of invaded J2 over a 7-day period. Overall, our study suggests that microbes that attach to J2 in soil can antagonize nematodes by ISR in plants. The microbes themselves induced plant responses, so it seems unlikely that the microbial attachment alters the nematode surface, thereby indirectly triggering ISR in the plant. However, this needs further investigations. It has been suggested that the intensity and the duration of ROS burst in plants can determine the compatible or incompatible interaction with RKN (Melillo et al., 2011; Sato et al., 2019). A strong and a prolonged ROS burst caused an incompatible interaction between the plant and RKN, based on an increased plant defense against J2 penetration (Melillo et al., 2011). In our study, we investigated whether the two *M. hapla*-isolated bacterial strains, K6 and E1 induce or increase ROS burst in tomato plants exposed to *M. hapla* J2, and whether the released compounds of the studied organisms induced a different reaction. In the ROS assay, the reaction of the plant was 100 times stronger in leaves than in roots. Moreover, the compounds released from the surface of J2 triggered a ROS burst. Likewise, Mendy et al. (2017) reported a strong induction of ROS burst in response to compounds released by two nematode species, *Heterodera schachtii* and *M. incognita*, which was accompanied by the expression of JA biosynthesis and signaling genes during the migratory phase in *A. thaliana.* In our study, K6 cells and cell-free compounds of both bacteria induced a much stronger ROS burst than the nematodes. Notably, the most prolonged ROS burst was recorded in the presence of compounds from J2 and strain K6 together, which might be evidence that active compounds are formed by an interaction of both organisms, for instance by degradation of the cuticle or J2-induced metabolites of strains K6 and E1.

## Conclusions

Nematodes encounter a vast number of microorganisms in soil but specifically attach only a few of them (Adam et al., 2014b; Elhady et al., 2017). However, the antagonistic properties of nematode-attached microbes have been hardly investigated, with the exception of *Pasteuria* endospores. Recently, we have isolated bacterial strains that attached to the cuticle of J2 of *M. hapla* in different soils and demonstrated their effects against J2 viability and invasion into roots (Topalović et al., 2019). Our current study aimed to investigate whether the nematode-attached microbes can trigger ISR in plants and in this way contribute to soil suppressiveness against *M. hapla*. The results suggest that the microbes attaching to *M. hapla* J2 in suppressive Geisenheim soil triggered a differential expression of PTI-responsive genes in tomato roots, 3 dpi. In addition, it was also shown that the attachment of *Microbacterium* sp. K6 to J2 prior to their invasion into the roots induced expression of PTI-like genes in roots and leaves. The attachment of K6 cells to the J2 significantly reduced the J2 establishment, confirming a plant-mediated antagonistic effect of strain K6 against *M. hapla* (Topalović et al., 2019). Overall, our data suggest that nematode-attached microbes have a positive role in plant defense against the sedentary endoparasite *M. hapla*. Our study contributes to the current knowledge on the tripartite plant-nematode-microbe interactions in soil and on nematode suppression by microbially induced systemic resistance in plants.

## Data Availability Statement

All datasets generated for this study are included in the article/**Supplementary Material**.

## Author Contributions

OT did the experiments, contributed to the ideas, and wrote the manuscript. SB and AS designed and performed the ROS assay together with OT. OT and HH statistically analyzed the data. HH initiated and supervised the research, and revised the manuscript. All authors read, revised, and approved the final manuscript.

## Funding

The study was funded by the German Research Foundation grant DFG HE6957/1-1.

## Conflict of Interest

The authors declare that the research was conducted in the absence of any commercial or financial relationships that could be construed as a potential conflict of interest.
