# Supplementary material for: Microbes Attaching to Endoparasitic Phytonematodes in Soil Trigger Plant Defense Upon Root Penetration by the Nematode
Source: Front Plant Sci. 2020 Feb 25;11:138. doi: 10.3389/fpls.2020.00138 (PMC7052486; doi:10.3389/fpls.2020.00138)
Supplement: Supplementary file 1 [file DataSheet_1.docx]

Supplementary Material

Microbes attaching to endoparasitic phytonematodes in soil trigger plant defence upon root penetration by the nematode

**Olivera Topalović^1^, Sandra Bredenbruch^2^, A. Sylvia S. Schleker^2^, Holger Heuer^1*^**

^1^ Dept. Epidemiology and Pathogen Diagnostics, Julius Kühn-Institut — Federal Research Centre for Cultivated Plants, Braunschweig, Germany

^2^ Rheinische Friedrich-Wilhelms-University of Bonn, INRES – Molecular Phytomedicine, Bonn, Germany

*** Correspondence:**

Dr. Holger Heuer
[holger.heuer@julius-kuehn.de](mailto:holger.heuer@julius-kuehn.de)

**
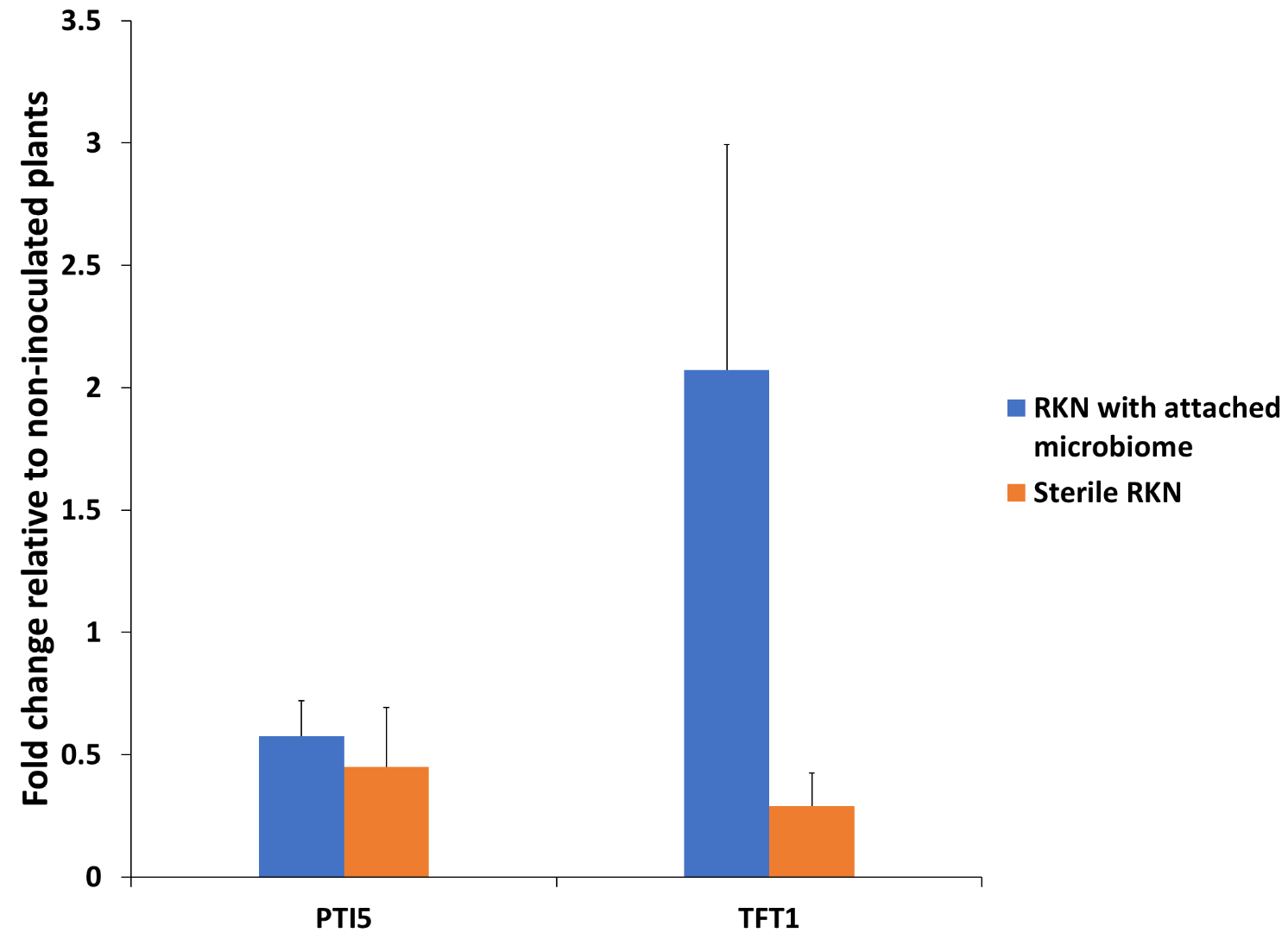
**

**Supplementary Figure S1.** Change in the expression of defence genes in tomato roots in response to invading root-knot nematodes (RKN) with or without microbes that adhered to the cuticle of second-stage juveniles (J2) of *Meloidogyne hapla*. Surface-sterilized J2 were incubated overnight in a suspension of a suppressive soil from a greenhouse in Geisenheim, or in sterile tap water as a control. To remove loosely attached microbes, J2 were extensively washed with sterile tap water and inoculated to 2-week old tomato plants grown in Petri dishes with MS-Gelrite medium. Five replicate plants were inoculated in each treatment. The roots were harvested for RNA extraction 3 days after nematode inoculation. Error bars show SE. Differences among treatments were not significant (*P* < 0.05, Tukey test on log-transformed data).

**Supplementary Figure S2.** Root ROS response of *S. lycopersicum* to second-stage juveniles of *Meloidogyne hapla* or their surface-released compounds (NemaWater) alone or with associated bacteria (K6, E1). Positive control is Flg22. Mean + SD of three biological replicates and n=12.

**Supplementary Figure S3.** Root ROS response of *S. lycopersicum* to second-stage juveniles of *Meloidogyne hapla* or their surface-released compounds (NemaWater) alone or with associated bacteria (K6, E1). The sum of relative luminescence units over two hours in an ROS assay with tomato leaves is shown. Positive control is Flg22. Mean + SD of three biological replicates and n=12. Different letters above bars indicate significant differences (*P* < 0.05, Student’s t-tests).

**Supplement Statistics1.** Analysis of varience of defence gene expression (factor gene: *GRAS4.1*, *MPK1*, *PDF1.2*, *PR1a1*, *TFT1*, *WRKY28*; log transformed data of fold change relative to plants that were not invaded by *Meloidogyne hapla* J2) after root invasion of *M. hapla* J2, as affected by attachment of microbes from soil G to the cuticle of J2 (factor microbiome: 1, 0), using generalized linear models and statistics software SAS 9.4.

Script:

data plantresponse; /* dataset */

input gene $ microbiome logfold;

cards;

GRAS4.1 1 0.333162521

GRAS4.1 1 0.723879454

...

WRKY28 0 -0.080482717

WRKY28 0 -0.139180124

;

ODS graphics on;

proc glm data = plantresponse plots=diagnostics; /* Generalized Linear Model */

class gene microbiome; /* Class variables */

model logfold = gene microbiome gene * microbiome; /* Model statement */

run;

ODS graphics off;

Output:


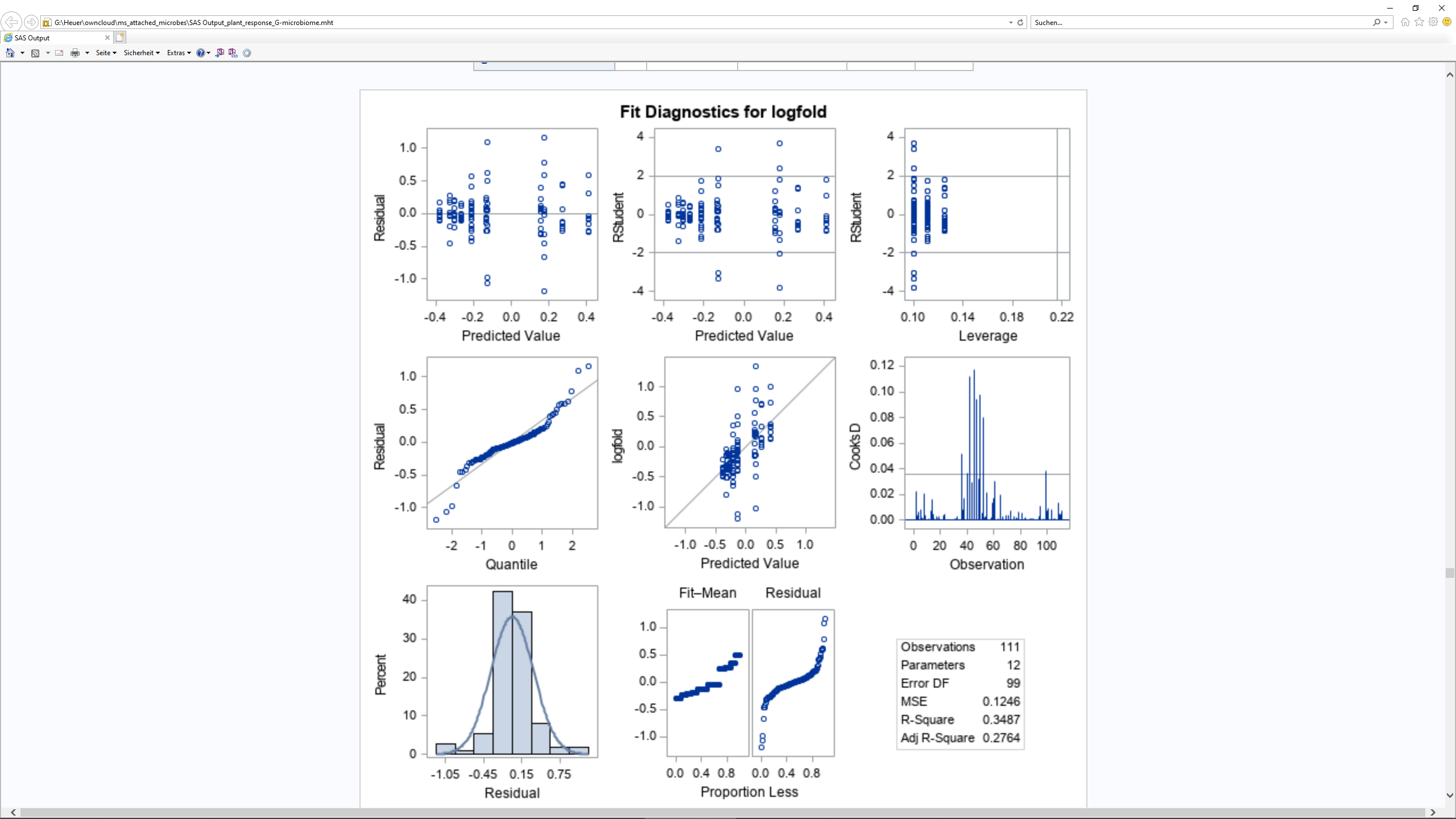

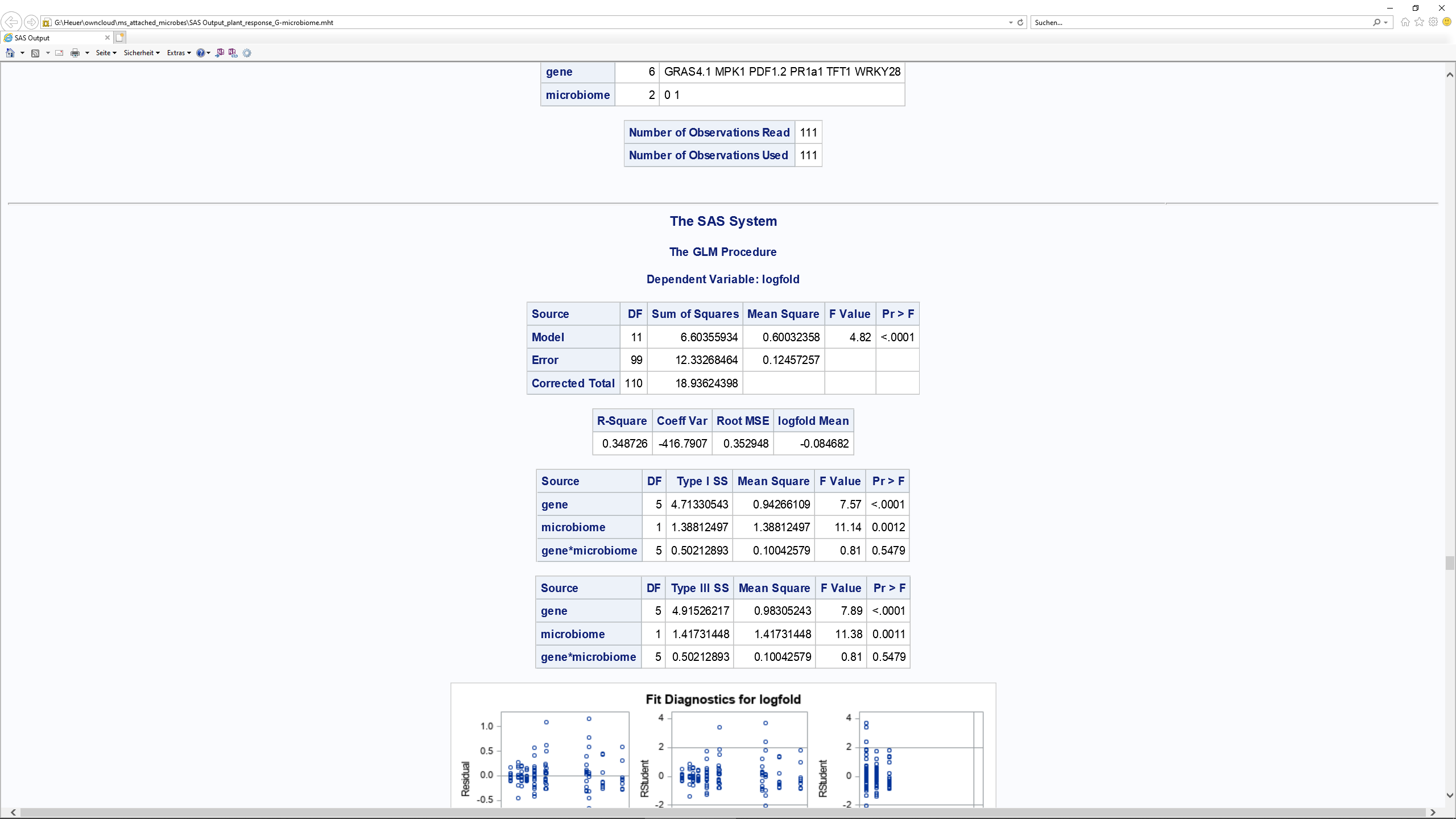


**Supplement Statistics2.** Analysis of varience of defence gene expression (dependent variable logfold: log transformed data of fold change relative to plants that were not invaded by *Meloidogyne hapla* J2) of PTI defence genes (factor gene: *GRAS4.1*, *MPK1*, *PDF1.2*, *PR1a1*, *TFT1*, *WRKY28*) in roots and leaves (factor tissue) after inoculation (factor dpi: 1, 3 days) and root invasion of *M. hapla* J2, as affected by attachment of cells of the bacterial strain *Microbacterium* sp. K6 to the cuticle of J2 (factor K6: 1, 0), using generalized linear models and statistics software SAS 9.4.

A) Overall effects

Script:

data plant_defence_K6;

input tissue $ gene $ dpi K6 logfold;

cards;

leaf GRAS4.1 1 1 -0.04624306

leaf GRAS4.1 1 1 -0.10798123

...

root WRKY28 3 0 0.18399956

root WRKY28 3 0 0.42509098

;

ODS graphics on;

proc glm data= plant_defence_K6 plots=diagnostics;


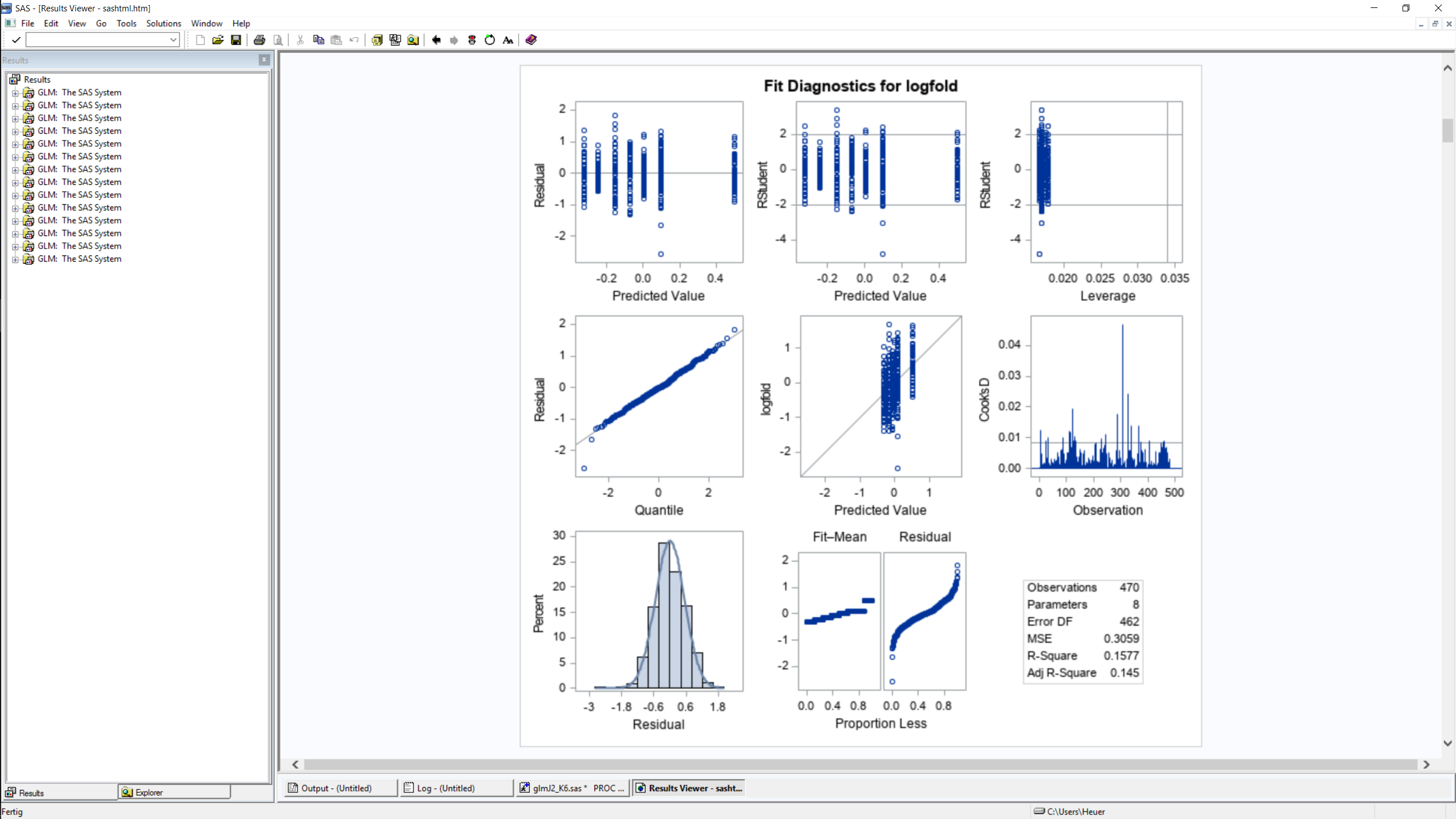
 class K6 gene dpi tissue;

model logfold = K6 | dpi | tissue | gene;

run;

Output:


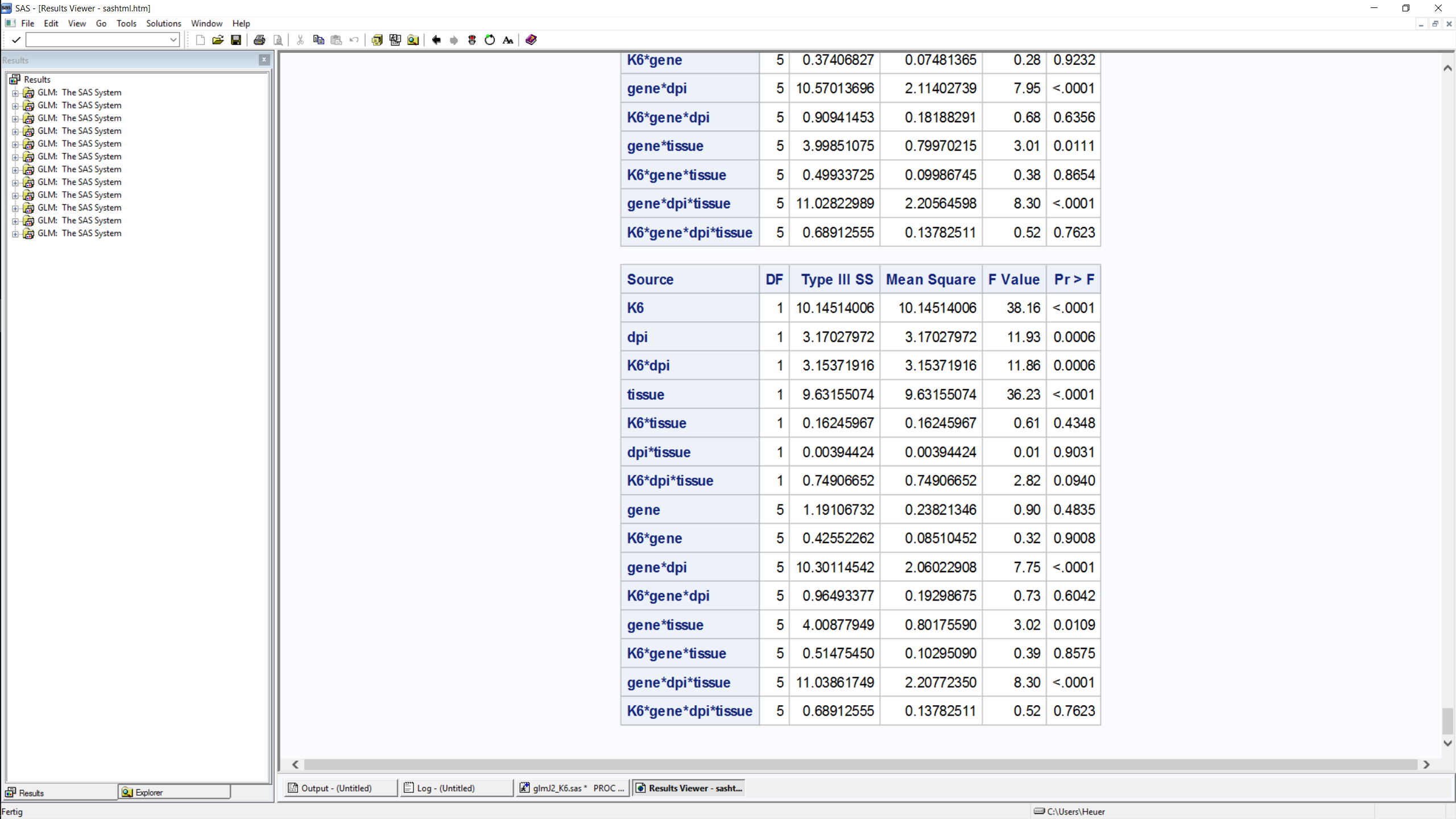


B) After backward elimination of non-significant factors and interactions

Script:

proc glm data= plant_defence_K6 plots=diagnostics;

class K6 gene dpi tissue;

model logfold = K6 dpi tissue K6*dpi;

run;

Output:


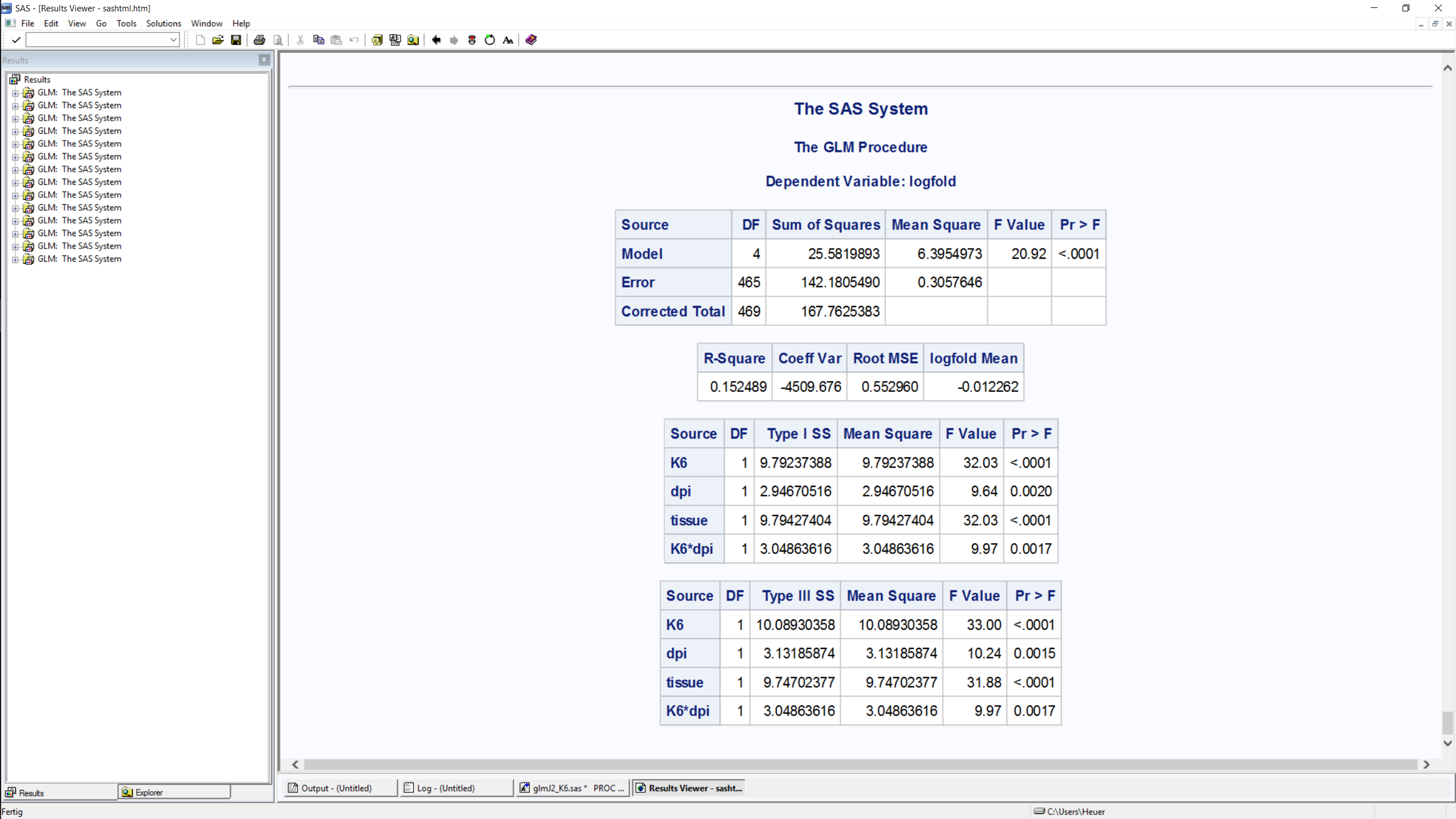


C) GLM analysis for each tissue and dpi:

| Tissue | dpi | P(factor K6) |
| --- | --- | --- |
| Root | 1 | 0.0724 |
| Root | 3 | 0.0001 |
| Leaf | 1 | 0.3920 |
| Leaf | 3 | 0.0001 |

D) Pairwise comparisons for significant differences between plants after invasion of nematodes with or without microbes attached to the cuticle, by tissue, gene, and dpi.

Script:

proc sort data=J2K6 out=K6sort; by tissue gene dpi;

proc genmod data=K6sort; by tissue gene dpi;

class K6;

model logfold = K6 / type3 scale=pearson;

run;
